# Senescence-Specific Expression of *RAmy1A* Accelerates Non-structural Carbohydrate Remobilization and Grain Filling in Rice (*Oryza sativa* L.)

**DOI:** 10.3389/fpls.2021.647574

**Published:** 2021-04-27

**Authors:** Ning Ouyang, Xuewu Sun, Yanning Tan, Zhizhong Sun, Dong Yu, Hai Liu, Citao Liu, Ling Liu, Lu Jin, Bingran Zhao, Dingyang Yuan, Meijuan Duan

**Affiliations:** ^1^Longping Branch of Graduate School, Hunan University, Changsha, China; ^2^College of Agronomy, Hunan Agricultural University, Changsha, China; ^3^State Key Laboratory of Hybrid Rice, Hunan Hybrid Rice Research Center, Changsha, China

**Keywords:** rice grain filling, NSCs transport, leaf starch, *RAmy1A*, specific-expressing

## Abstract

Remobilization of pre-anthesis NSCs (non-structural carbohydrates) is significant for effective grain filling in rice (*Oryza sativa* L.). However, abundant starch particles as an important component of NSCs are still present in the leaf sheath and stem at the late stage of grain filling. There are no studies on how bioengineering techniques can be used to improve the efficiency of NSC remobilization. In this study, *RAmy1A* was expressed under the senescence-specific promoter of *SAG12*, which was designed to degrade starch in the leaf sheath and stem during grain filling. *RAmy1A* mRNA successfully accumulated in the leaf, stem, and sheath of transgenic plants after anthesis. At the same time, the starch and total soluble sugar content in the leaf, stem, and leaf sheath were obviously decreased during the grain-filling period. The photosynthetic rate of transgenic lines was higher than that of the wild types by an average of 4.0 and 9.9%, at 5 and 10 days after flowering, respectively. In addition, the grain-filling rate of transgenic lines was faster than that of the wild types by an average of 26.09%. These results indicate an enhanced transport efficiency of NSCs from source tissues in transgenic rice. Transgenic rice also displayed accelerated leaf senescence, which was hypothesized to contribute to decreased grain weight.

## Introduction

Grain filling is a significant and dynamic process for the final yield of rice. Previous studies have shown that the assimilates required for grain filling are primarily supplied by leaf photosynthesis and non-structural carbohydrates (NSCs) reserved in leaf sheaths and stems before anthesis (Schnyder, 1993; Samonte et al., 2001; Xie et al., 2015; Zakari et al., 2020). NSCs can contribute to 10–40% of final grain yield, depending on the variety and environmental conditions (Takai et al., 2005; Yang and Zhang, 2010). Conversely, NSCs reserved in the stems could also enhance sink strength during grain filling and increase the filling efficiency (Yang and Zhang, 2010; Fu et al., 2011). In several rice varieties, a large amount of NSCs were not transported to the grains during the later filling period (Yang et al., 2002). Thus, breeders can develop high-yielding rice varieties by improving the transport efficiency of reserved NSCs in leaf sheaths and stems (Takai et al., 2005).

Starch can be synthesized and stored in chloroplasts and amyloplasts from photosynthetic and non-photosynthetic cells, respectively, as an important component of NSCs. In leaves, a fraction of the carbon assimilated through photosynthesis is converted to transitory starch in chloroplasts during the day, which is degraded at night to provide substrates for consumption and morphogenesis of the whole plant (Zeeman et al., 2010). A portion of the photoassimilates are translocated to the rice stem and stored as starch during the vegetative stage, when excess photoassimilates are available. Starch and other NSCs stored during this progress will subsequently be degraded and transported into the kernel, supporting leaf photosynthesis during grain filling (Slewinski, 2012; Wang et al., 2016). The endoamylase α-amylase, which hydrolyzes α-1, 4-glycosidic bonds, plays an important role in the first step of starch degradation in such processes (Zeeman et al., 2010). Previous studies have also demonstrated a positive relationship between starch degradation and alpha-amylase (EC 3.2.1.1) activity (Sugimura et al., 2015).

Rice α-amylase has several isoforms, encoded by a multigene family. Among them, more than 20 native α-amylase isoforms have been identified and characterized (Asatsuma et al., 2005; Njeri et al., 2019). These isolated α-amylase isoforms are mostly encoded by *RAmy1A*, R*Amy3B, RAmy3C, RAmy3D*, and *RAmy3E* (Nanjo et al., 2004; Nakata et al., 2017). *RAmy1A* and *RAmy3E* were expressed in all tissues, while a weak expression of *RAmy1B* was detected only in the callus (Huang et al., 1991). The *RAmy1A* product favored both soluble starch and starch granules, while the *RAmy3D* product had a high affinity for maltoheptaose as well as soluble starch (Asatsuma et al., 2005). A high expression of *RAmy1A* could lead to higher α-amylase activity and defective starch granule development (Zhang et al., 2008). A previous study has also demonstrated that the half-life of *RAmy3D* and *RAmy3E* mRNAs was substantially shorter than that of *RAmy1A* in cultured rice suspension cells, regardless of the presence or absence of sucrose in the medium (Sheu et al., 1996).

Several physiologists and breeders propose that improving the remobilization and transport efficiency of NSCs stored in rice source organs during vegetative growth could be an efficient way to improve the rice grain-filling rate and yield (Fu et al., 2011; Wang et al., 2016; Zakari et al., 2020). However, most of the previous studies have focused on adjusting traditional cropping strategies (Pan et al., 2010; Chen et al., 2019). For example, Wei et al. (2004) found that mild drying of the soil 9 days after rice anthesis inducedα-amylase activity, and effectively promoted the remobilization of stored NSCs in stems, and subsequently reduced the yield loss caused by unfavorable-delayed senescence. However, little is known about how bioengineering techniques can be used to improve germplasm in this field. We hypothesized that increasing *RAmy1A* gene expression at the rice grain-filling stage will aid degradation of stored starch into soluble sugar, thus enhancing the NSC supply for grain filling. To verify this, rice plants were transformed with *RAmy1A* CDS, guided by the promoter of Arabidopsis *SAG12*, which was specifically expressed during the senescence stage (Shan et al., 2019), using an *Agrobacterium tumefaciens*-mediated transformation method. This senescence-specific expression of *RAmy1A* may minimize its effects on vegetative growth. The phenotype for the transgenic lines were then analyzed. Gene expression, α-amylase activity, starch and soluble sugar content in leaf, stem, and leaf sheath were investigated. The photosynthesis rate (Pn) of the rice leaf was also evaluated at different growth phases. This study provides insights into accelerating rice grain filling and enhancing NSC remobilization through molecular biology techniques.

## Materials and Methods

### Generation of *RAmy1A* Expression Vector

**Figure 1:**
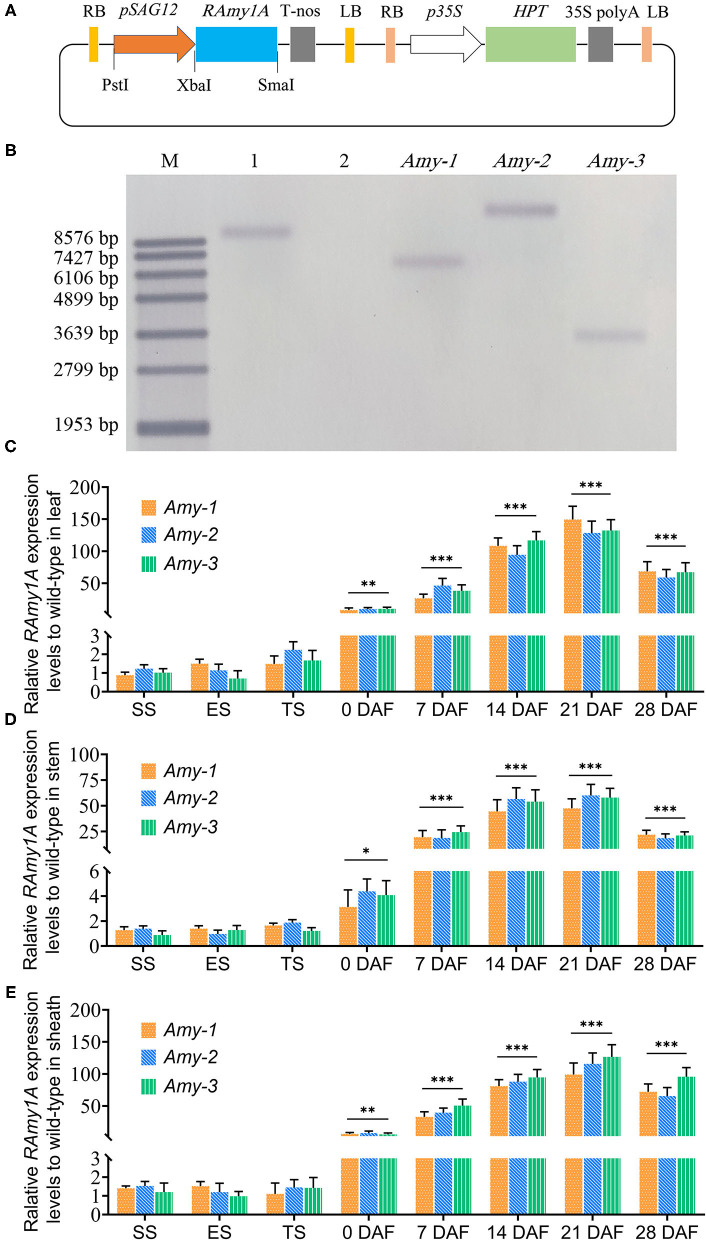
Construction of the plant expression vector *pSAG12::RAmy1A* and molecular detection of transgenic rice lines. **(A)** Schematic diagram of the plasmid constructions. **(B)** Southern blot analysis of *RAmy1A* gene transgenic lines. M—DNA Molecular Weight Marker VII, DIG-labeled; 1—genomic DNA from plasmid as a positive control; 2—genomic DNA from the wild type rice plants as a negative control; and *Amy-1, Amy-2*, and *Amy-3* are genomic DNA from transgenic lines we chose for the study. The genomic DNA was digested with BamHI and hybridized with a *pSAG12*-specific probe labeled with digoxigenin. **(C**–**E)** Relative expression of *RAmy1A* gene to wild types measured by quantitative real-time PCR. SS-seedling stage, ES-elongation stage, TS-tillering stage, DAF-day after flowering. *P < 0.05 vs. WT, **P < 0.01 vs. WT, and ***P < 0.001 vs. WT.

The full-length coding sequence (CDS) was amplified by polymerase chain reaction (PCR) from rice (Xianghui 299, *Indica*) cDNA to construct the vector for senescence-specific expression of *RAmy1A* (LOC_Os02g52710), using the forward primer 5′-CTCTAGAGCATGCAGGTGCTGAACA-3′ and the reverse primer 5′-CCCGGGTCGTGGACAATTGCATCC-3′, which contained the enzyme site of XbaI and SmaI, respectively. The promoter fragment of the *SAG12* gene (AT5G45890) was amplified from *Arabidopsis thaliana* genome with the forward primer, 5′-CCTGCAGGATATCTCTTTTTATATTCA-3′ and the reverse primer, 5′-CTCTAGAGGTTTTAGGAAAGTTAAATG-3′. Recognition sites for PstI and XbaI were added for each, respectively. The amplified fragments of the *RAmy1A* CDS and *pSAG12* fragment were then sequentially integrated into the pSB130M expression vector (Figure 1A). The sequence of the cloned *RAmy1A* gene and the promoter fragment of the *SAG12* gene are provided in the Supplementary Materials.

### Plant Materials and Transformation

Xianghui 299, an *indica* rice variety (developed by the Hunan Hybrid Rice Research Center, Changsha, China), was used as the wild type in this study, which was transformed with the constructed vector via the *Agrobacterium tumefaciens*-mediated approach (Yuan et al., 2016). Thereafter, five homozygous transgenic lines were selected from 19 positive transformants and three of them (the third generation:*Amy-1, Amy-2*, and *Amy-3*) were used for detailed studies.

Transgenic lines and the corresponding wild-type plants were experimentally cultivated in fields during the natural rice growing seasons in Changsha (28°21′N, 113°26′E) and Sanya cities (18°30′N, 109°51′E) in southern China (Supplementary Tables 1, 2). Three biological replicate samples of leaf, stem, and leaf sheath were taken at sunset during the seeding, tillering, heading, and maturation stages of the Changsha growth season. The samples were immediately frozen and stored in liquid nitrogen before biochemical indicators were measured. The fully expanded leaves of the wild type and transgenic plants were harvested in three biological replications at different growing stages (seedling, elongation, and tillering stages, 0 days after flowering (0 DAF), 7, 14, 21, and 28DAF) for real-time quantitative PCR.

### Southern Blot and Gene Expression Analysis

For southern blot, leaf genomic DNA was extracted using the CTAB method and purified with the EasyPure® Plant Genomic DNA Kit (Transgenbiotech, #EE111-01, China). Thereafter, 5 μg of purified genomic DNA was digested with BamHI and separated by electrophoresis in a 1% agarose gel and then transferred to a Hybond N+ nylon membrane (Amersham, #RPN303B, UK). The *pSAG12*-specific probe (950 bp) was labeled with DIG-dUTP in PCR, using the PCR DIG Probe Synthesis Kit (Roche, # 11636090910, Switzerland). Subsequent hybridization was performed using the DIG High Prime DNA Labeling and Detection Starter Kit I (Roche, # 11745832910, Switzerland) according to standard protocols.

Gene expression level was detected using a q-RT PCR assay. Total RNA was extracted using the TriZol reagent (Invitrogen, #15596026, Germany) and treated with DNase I (Thermo, #EN0521, USA) to avoid genomic DNA contamination. First-strand cDNA was synthesized from each preparation of total RNA (1 μg/reaction) using the RevertAid First-Strand cDNA Synthesis Kit (Thermo Fisher Scientific, #K1622, USA) and an oligo-dT primer. Real-time quantitative PCR was performed using a LightCycler® 480 II system (Roche) with PowerUp™ SYBR® Green Master Mix (Thermo Fisher Scientific, #A25742, USA) in accordance with the manufacturer's instructions. *UBQ5* was used as the internal control gene with the forward primers 5′-GTATTCCACCTGTTCAGC-3′ and the reverse primers 5′-ACCTTCAATGTTGTAATCCTT-3′. The forward and reverse primers for the *RAmy1A* gene were 5′-TCCTCATCGTCCTCCTTG-3′ and 5′-ACTCCCAGTTGAATCCCT-3′, respectively.

### Measurements of the Endosperm and Grain Weight

To compare the increase in endosperm and grain weight between transgenic and wild types, freshly opened (0 DAF) spikelets were labeled with marker pens. At 3, 5, 7, 9, 11, 13, 15, 17, 19, 21, 23, 25, 27, 29, 31, 33, and 35 DAF, 30 developing grains were collected to determine the fresh and dry weight of the endosperm, which was gently lifted from developing grain using a fine forceps. The resulting endosperm samples were weighed immediately with a high-precision electronic scale (Sartorius, #CPA225D, Germany) to determine the fresh weight. The of a single endosperm was calculated accordingly. The dry weight of endosperm samples was measured after they were oven dried at 70°C for 24 h. The 1,000-grain weight was measured using filled grains after maturation.

### Amylase Activity Assay and the Measurement of Starch and Soluble Sugar Content

The α-amylase Assay Kit (Solarbio, #BC4570, China) was used to test for α-amylase activity, according to the manufacturer's instructions. The starch content was measured with the Starch Content Assay Kit (Solarbio, #BC0700, China). Soluble sugar was separated from starch using 80% ethanol. The acid hydrolysis method was then used to decompose starch into glucose, and the anthrone colorimetric method was used to determine glucose levels, which were then converted to starch content. Quantitative analyses of carbohydrates were performed using the Plant Soluble Sugar Content Assay Kit (Solarbio, #BC0035, China). The reducing ratio (%) of starch content between the heading and the harvest was calculated using the following formula:

$$=\frac{\begin{array}{c} \text{Reducing ratio of starch content during grain filling }(\text{\%})\\ \text{The difference of sarch content }\\ \text{between heading and harvest stages } \end{array}}{\text{ The starch content at heading stage }}\times 100$$

The same calculation method was applied to the reducing ratio of soluble sugar concentration from heading to harvest.

### Measurement of Net Photosynthetic Rate in Rice Leaves

The net photosynthesis rate (Pn) of the fully expanded leaf was measured between 9:00 and 11:00 a.m., using an LI-6800 portable photosynthesis system (LI-COR, Lincoln, NE, USA). The following settings were used for the leaf chamber condition: flow rate of 500 μmol·s^−1^; ΔP auto of 0.2 kPa; RH_air of 60%; CO_2_ reference of 400 μmol·mol^−1^; mixing fan speed of 10,000 rpm; temperature Txchg of 31°C; and PAR of 1,400 μmol·m^−2^·s^−1^. The Pn was adjusted according to actual leaf area during the measurement. Four biological replicates were used.

## Results

### The *pSAG12* Enhanced *RAmy1A* Expression During the Post-anthesis Stage

The successful construction of the *pSAG12*::*RAmy1A* vector (Figure 1A) was determined by gene sequencing (data not shown). Southern blot analysis showed the three *RAmy1A*—overexpression (OE) lines all had one copy of the target gene (Figure 1B). The expression level of *RAmy1A* in T_3_ plants was considerably enhanced in transgenic lines after flowering (Figures 1C–E). No significant increase in *RAmy1A* expression was observed in transgenic lines, compared with the wild types at the vegetative stage (seedling, elongation, and tillering), while at the reproductive stage, the transcription level of *RAmy1A* in transgenic lines was strongly increased compared with the wild type, and the induction in the leaf tissue was 128 times greater at 21 DAF (Figure 1C). The expression of *RAmy1A* continued to increase in the leaf, stem, and leaf sheath from 0–21 DAF, but was lower on 28 DAF than on 21 DAF (Figures 1C–E).

### *RAmy1A*-Overexpression Accelerated Grain Filling Rate and Shortened Reproductive Growth Period

**Figure 2:**
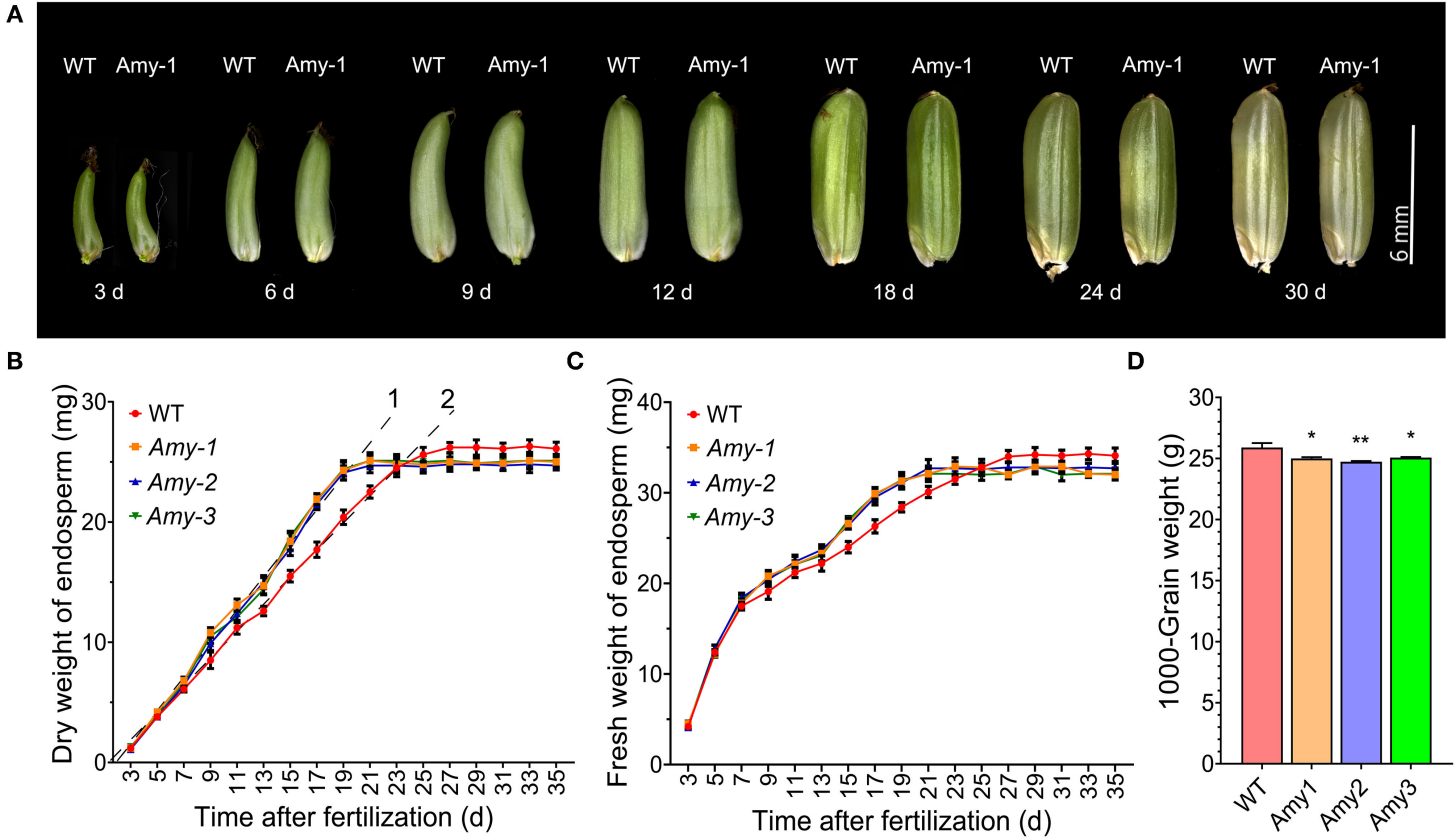
The changes in endosperm weight and grain-filling rate characteristics during the grain filling period. **(A)** Growth and the change of grain morphologies. Scale bar = 6 mm. **(B,C)** Dry and fresh weight of endosperm changes. **(D)** 1,000-Grain weight of the transgenic lines and WT. Values are given as the mean ± SD. *P < 0.05; **P < 0.01 compared with the wild type using Student's *t*-test.

As *RAmy1A* encodes α-amylase, which can attach to intact starch granules and plays a significant role in starch breakdown, *RAmy1A*-overexpression most likely affects the NSC utilization levels and the grain-filling rate in rice. We compared the dry and fresh weight of the endosperm of transgenic and wild types at the reproductive stage (Figure 2). Different endosperm weights were observed for the OE lines and wild types after seven DAF. The endosperm weight of transgenic plants stopped increasing at 21 DAF, while in the wild type, it was still increasing until 27 DAF. Thus, grain filling of *RAmy1A*-OE lines was completed earlier than in the wild types. The grain-filling rate in OE lines between 7 and 19 DAF (1.45 mg/d) was accelerated compared with the wild type which increased dry matter by 1.15 mg/d (Figure 2B). At maturation, the 1,000-grain weight of OE lines was less than that of the wild types by 0.9 g on average (Figure 2D), while the grain shape was similar during grain development (Figure 2A). The number of grains per panicle and the seed-setting rates were not significantly different for the OE lines (Supplementary Figure 1).

**Figure 3:**
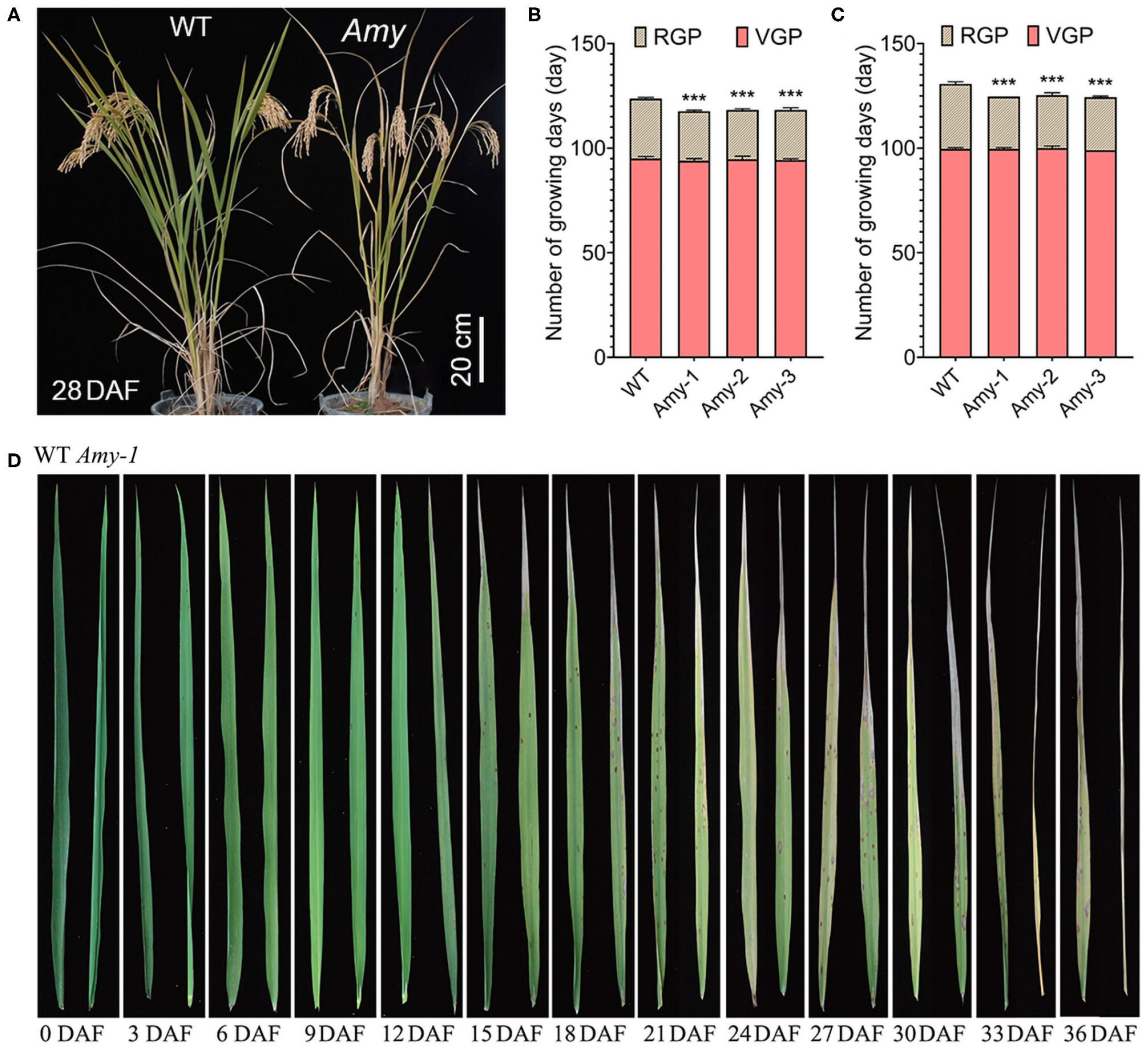
Rice plant morphology at harvest and the change of blade leave after flowering. **(A)** Rice plant morphology at 28DAF. **(B)** Number of rice growth days in the summer season at Changsha. **(C)** Number of rice growth days in the winter season at Sanya. RGP-reproductive growth period, VGP-vegetative growth period. Values are given as the mean ± SD. ***P < 0.001 compared with the wild type using Student's *t*-test. **(D)** Changes in rice leaves after flowering. For each DAF, left for WT leaf and right for *Amy-1* leaf.

The vegetative growth period of *RAmy1A*-OE lines was consistent with that of the wild types, while the reproductive growth period was shortened by 5 days on average (Figures 3B,C and Supplementary Tables 1, 2). Although the flag leaf shape did not change, leaf senescence was accelerated in the transgenic mutant during the grain-filling stage compared with the wild types (Figures 3A,D).

### Increased α-Amylase Activity and Decreased Starch Content in Stems, Leaf Sheaths, and Leaves of *RAmy1A*-OE Lines

**Figure 4:**
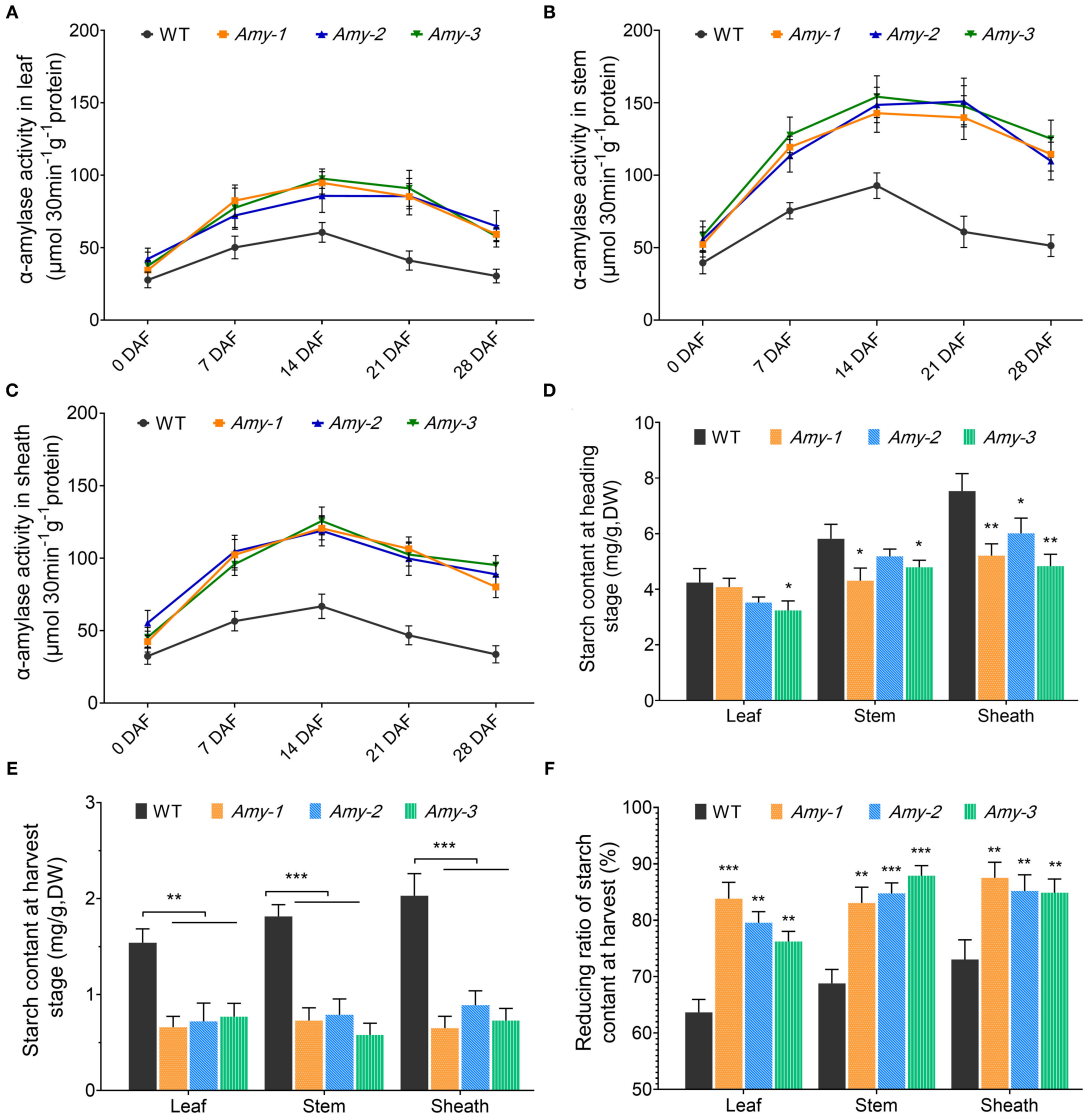
α-amylase activity and starch content in source tissues. **(A**–**C)** α-amylase activity in rice leaf, stem, and leaf sheath at the grain filling stage. **(D,E)** Starch content in source tissues at the heading and harvest stages. **(F)** Reducing ratio of starch content in source tissues at harvest. Asterisks indicate significant differences compared with WT, as determined by Student's *t*-test. *P < 0.05; **P < 0.01; ***P < 0.001. Bars indicate standard deviations in three independent plants.

We measured the α-amylase activity in stems, leaf sheaths, and leaves at 0, 7, 14, 21, and 28 DAF, together with starch content at the heading and harvest stages to verify whether *RAmy1A* OE enhanced the α-amylase activity and affected the starch content in the chlorenchyma. As shown in Figure 4, α-amylase activity increased continuously from 0 DAF to 14 DAF in both *RAmy1A*-OE lines and wild types. In three *RAmy1A*-OE lines (*Amy1, Amy2*, and *Amy3*), amylase activity was markedly increased compared with the wild types in leaves, stems, and leaf sheaths after flowering. α-amylase activity in the stem of the wild type at 28 DAF decreased from that at 0 DAF, while it still remained high in transgenic plants at 28 DAF (Figure 4B). A similar pattern was observed in the leaf (Figure 4A). The maximum difference in α-amylase activity in the leaves and stems between transgenic and wild types occurred at 21 DAF, with an increase to 208 and 238%, respectively. However, α-amylase activity in leaf sheaths in the *RAmy1A*-OE lines maintained a high level after seven DAF, compared to the wild types. The starch content in the chlorenchyma of the three *RAmy1A*-OE lines decreased significantly compared with the wild types, especially at the harvest stage. The starch content in the leaves, stems, and leaf sheaths of the *RAmy1A*-OE lines was, on average, 46, 38, and 37%, respectively, that of the wild types. We further calculated the decreasing rate of starch content during grain filling, which, to some extent, represents the starch transferral rate. The results suggest that *RAmy1A*-specific overexpression significantly enhanced starch remobilization in leaves, stems, and leaf sheath in the *RAmy1A*-OE lines, compared with the wild types (Figure 4F).

### Decreased Soluble Sugar Content in the Stems, Leaf Sheaths, and Leaves of *RAmy1A*-OE Lines

**Figure 5:**
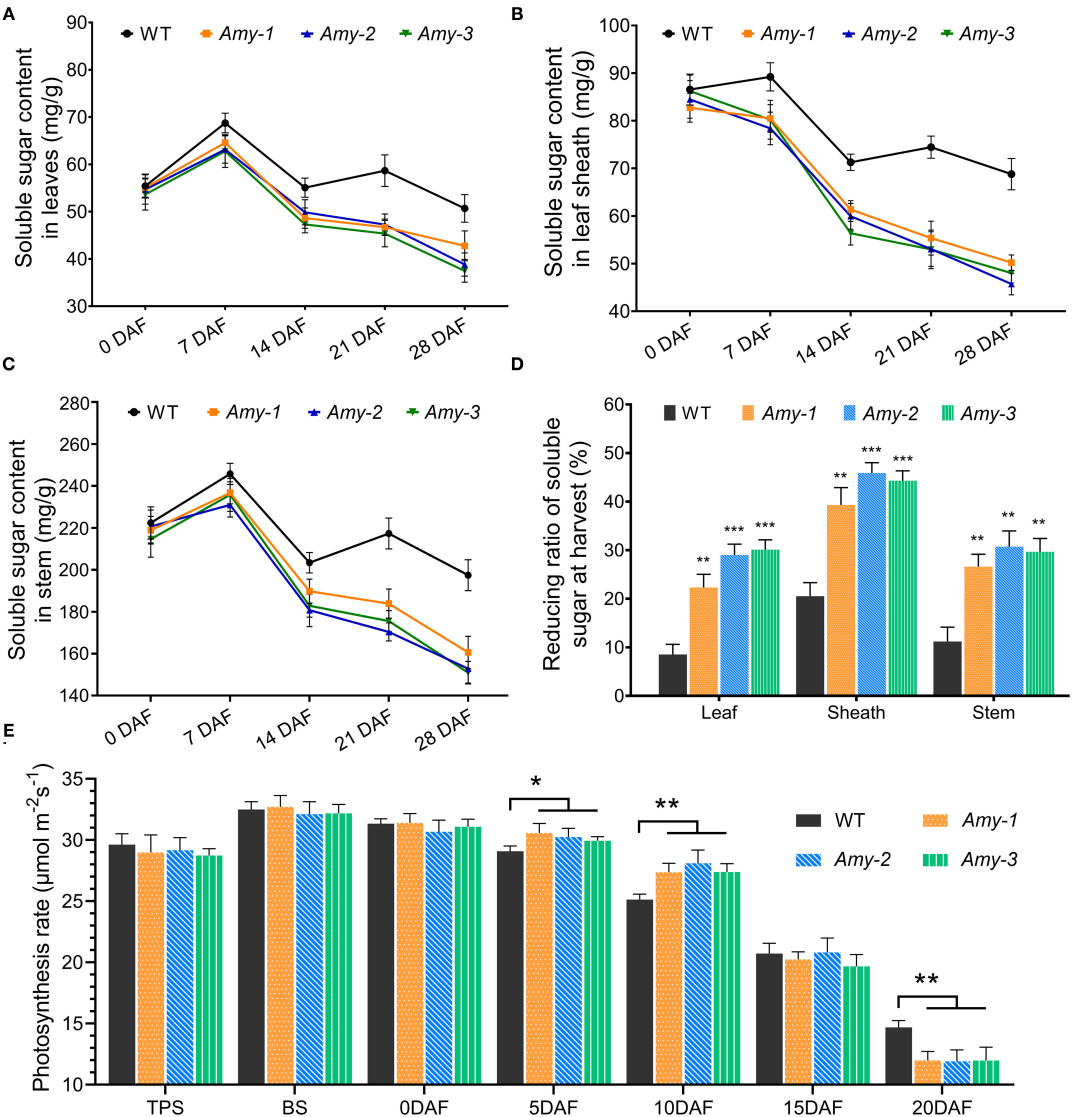
Soluble sugar content in stems, leaf sheaths, and leaves of transgenic rice plants and the Pn of rice plants. **(A**–**C)** Soluble sugar content in rice leaf, stem, and leaf sheath at the grain-filling stage. **(D)** Reducing ratio of soluble sugar content in source tissues at harvest. **(E)** Rice leaf Pn at different growth stages; namely the TPS-top tilling stage, BS-booting stage, DAF-day after flowering. Asterisks indicate significant differences compared with WT, as determined by Student's *t*-test. *P < 0.05; **P < 0.01; ***P < 0.001. Bars indicate standard deviations of at least three independent plants.

As shown in Figure 5, total soluble sugar content in the leaf increased from 0 to 7 DAF and decreased from 7 to 14 DAF for both *RAmy1A*-OE lines and wild types. The highest levels were observed at 7 DAF. The soluble sugar content varied between 14 and 28 DAF. The levels increased from 14 to 21 DAF and then decreased from 21 to 28 DAF in the wild types, while in transgenic lines, a continuous decrease was observed from 7–DAF (Figure 5A). The pattern of change in the soluble sugar content in the stem over time was consistent with that in the leaf (Figure 5C). However, the total soluble sugar contentment in the stem was about three times more than that in leaf. This is not the case in the leaf sheath, where the soluble sugar contentment continued to decrease during the grain-filling period in the transgenic lines. The results suggest that the *RAmy1A* specific overexpression dramatically enhanced the transport efficiency of soluble sugar out of the source tissues (Figure 5D).

### *RAmy1A* Overexpression Helps Maintain a High Photosynthesis Rate in Rice Plants

Starch is one of the major primary products of photosynthesis in the leaves of higher plants. We investigated whether the reduction of starch in the source leaves affected the Pn in rice. To this end, the Pn was measured at different growth periods. It is interesting to note that a significant decrease in NSC content in leaves helped rice plants maintain a relatively higher Pn after flowering. The highest Pn was observed at the booting stage for the wild types, which slowly decreased during the grain-filling periods (Figure 5E. However, the Pn of *RAmy1A*-OE lines was considerably higher than that of the wild types, by an average of 4.0% and 9.9% at 5 and 10 DAF, respectively (*P* < 0.01 separately for all tests). At 20 DAF, Pn of the three *RAmy1A*-OE lines was lower than that of the wild types (Figure 5E).

## Discussion

Previous studies have demonstrated that NSCs accumulated in rice stems prior to heading play an important role in yield formation which can later be mobilized to supplement photosynthetic production during grain filling (Pan et al., 2010; Wang et al., 2016; Zakari et al., 2020). Pre-anthesis NSC reserves in the stem are also closely associated with the sink strength during grain-filling of rice (Fu et al., 2011). This suggests that high pre-anthesis NSCs reserves benefit final rice yield. As a principal component of NSCs, starch accumulation was affected by α-amylase. Overexpression of the α-amylase gene throughout the rice growth period under the control of the *Cauliflower mosaic* virus 35S promoter affected NSC accumulation during the vegetative growth period and thereby the dry weight of seeds and the field-level yield (Asatsuma et al., 2006). In the present study, however, a senescence-specific expression promoter, *pSAG12*, was used to drive the *RAmy1A* gene (Figure 1A). The expression of the *RAmy1A* gene in transgenic lines during the vegetative growth period did not shown any significant difference compared with the wild types (Figures 1C–E). Moreover, rice growth and NSC accumulation were not affected before flowering (Figure 5). This creates the preconditions for an efficient grain filling progress. Nevertheless, we initially proposed that *RAmy1A* would be overexpressed at later grain-filling stages rather than at the beginning of flowering.

In cereal crops, a faster grain-filling rate, earlier and longer grain filling, and faster grain water absorption and loss rates were linked to a higher grain weight (Xie et al., 2015). However, occasionally there is a negative relationship between the grain-filling rate and duration (Wang et al., 2008; González et al., 2014; Shi et al., 2017). For example, under drought conditions, the grain-filling rate in maize increased, but the duration shortened (Wang et al., 2019). In the present study, we observed a similar phenomenon of accelerated grain filling-rate and a shortened duration in *RAmy1A*-OE lines (Figure 2B). The grain filling completion time of the *RAmy1A*-OE plant was shortened by ~6 days compared with the wild types (Figures 2B,C). This leads to a shortened reproductive growth period and a reduced 1000-grain weight in transgenic rice (Figures 2D, 3B). This might be due in part to accelerated leaf senescence (Figure 3D). It was proposed that because *RAmy1A*-OE lines entered senescence phase earlier than wild types, and the Pn decreased after 15 DAF (Figure 5E), there was not sufficient photosynthetic product for grain filling, and the filling process then terminated earlier. However, this may not be entirely accurate. Nevertheless, it is worth noting that the kernels of *RAmy1A*-OE lines also seemed to reach maturity earlier than those of wild types (Figure 2A). The earlier ripening of seeds facilitates the rapid production of grain in agriculture.

During grain filling in cereal crops, senescence occurs naturally, involving the coordinated degradation of macromolecules and the reserve remobilization from senescing tissues into reproductive organs (Mattoo and Aharoni, 1988; Kong et al., 2015). It has previously been observed that chlorophyll, protein, and starch degradation occurred as senescence-characteristic processes in aging plants (Mattoo and Aharoni, 1988). However, the results of the present study showed that highly overexpression of *RAmy1A* during the grain-filling period could result in early senescence in rice leaves (Figure 3D). According to the results, overexpression of *RAmy1A* induced α-amylase activity and consequently decreased the starch content in stems, leaf sheaths, and leaves (Figures 4A–E). To our knowledge, there have been no reports on starch degradation causing premature leaf aging in rice. Thus, we speculate that there are two possible causes for this: (i) the low starch content in the leaf triggered the molecular and cellular events underlying aging or (ii) overexpression of *RAmy1A* initiated the senescence signaling pathway. Prior studies that have noted that premature aging of rice hinders improved of yield significantly (Großkinsky et al., 2018; Han et al., 2020). Our original hypothesis was that increased transport of NSCs reserved in leaves, leaf sheaths, and stems enhanced the final yield. It might be feasible to use another later-active promoter (Noh and Amasino, 1999) to control the level of expression of the *RAmy1A* gene in future studies. A relatively lower level of expression of *RAmy1A* may enhance a rapid grain-filling rate while not causing premature senescence.

Large panicle rice has a large sink capacity; however, the inferior spikelets filling is poor in these varieties due to asynchronous grain filling. The slow grain-filling rate and the low grain weight of inferior spikelet impact on grain yield. This has often been attributed to limited carbohydrate supply or weak sink activity (Wang et al., 2015; Okamura et al., 2018; Chen et al., 2019). The present study showed that starch content in source organs was decreased at the heading stage in *RAmy1A*-OE lines, compared with the wild types (Figure 4D). This indicated that the *RAmy1A* gene, initiated by the *SAG12* promoter, started to function and starch was degraded into soluble sugar, increasing the NSCs supply which accelerated the grain-filling rate (Figure 2B). Previous research has documented that NSCs can stimulate sink activity and initiate and promote grain filling at the initial phase of grain filling (Tsukaguchi et al., 1996). There is reason to believe that *RAmy1A* overexpression derived from the *SAG12* promoter may benefit the activation of sink activity and the initiation of grain filling at the beginning of the grain-filling stage. The results also showed that the reducing ratio of starch and soluble sugar in transgenic lines at harvest was significantly higher than that of the wild types (Figures 4F, 5D), suggesting that the remobilization of NSCs and the transport efficiency of *RAmy1A* temporal-overexpression were increased. Effective coordination of source capacity, carbohydrate transport efficiency (flow) and sink activity can increase rice yields (Chen et al., 2019). Consequently, we propose that increasing *RAmy1A* expression levels by means of the *SAG12* promoter could benefit the grain-filling rate in some large panicle rice and increase the yields. This however, requires further research.

**Figure 6:**
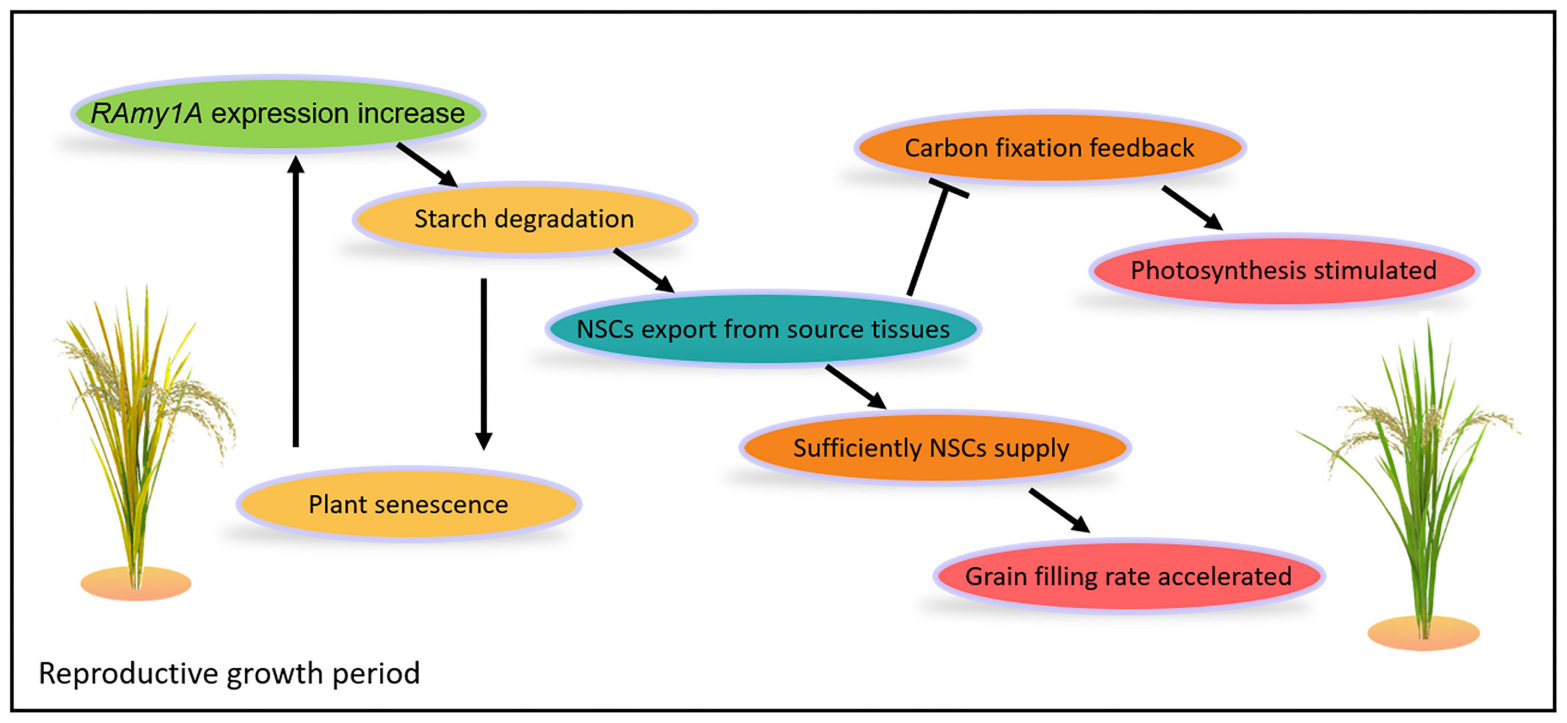
A hypothetical schematic diagram for the physiological processes of transgenic rice during the reproductive growth period. During the reproductive growth stage of transgenic rice, the expression level of *RAmy1A* gene was increased driven by *pSAG12*. This caused starch degradation, and the export of NSCs from the source tissues to fuel the grain filling process, thus leading to an accelerated grain-filling rate. In contrast, the exportation of NSCs decreased the negative sugar feedback on carbon fixation, resulting in a higher Pn. Plant senescence associated with starch degradation may stimulate *pSAG12* and further increase *RAmy1a* expression.

One unexpected result in this research was that the Pn of transgenic lines was relatively higher at 5 and 10 DAF than in the wild types (Figure 5E). In other words, *RAmy1A* overexpression helped rice plants maintain a relatively higher Pn during the mid-filling stage. Several reports have shown that sugar was involved in negative feedback on carbon fixation (Jang and Sheen, 1994). Increasing sucrose export rates could stimulate photosynthesis by decreasing this negative feedback (Bush, 2020; Xu et al., 2020). These outcomes thus result from decreased soluble sugar contentment and enhanced sugar transport efficiency in rice source tissues (Figure 5). The decreased Pn at 20 DAF could be attributed to accelerated leaf senescence in transgenic lines. A hypothetical schematic diagram for the physiological processes of transgenic rice in reproductive growth periods was produced, based on the results of the present study and previous studies (Figure 6).

## Conclusion

This study was evaluated the effect of senescence stage-specific overexpression of *RAmy1A* on grain filling in rice. Transgenic lines showed accelerated grain-filling rates, enhanced NSC transport efficiency and shortened growth periods, while maintaining a relatively higher Pn during the mid-filling stage. However, the disadvantageous trait of accelerated leaf senescence and decreased grain weight was also noted in the transgenic lines. We believe that controlling *RAmy1A* gene expression with a different senescence-specific promoter could prevent premature leaf aging and achieve a longer period of grain filling. This study offers valuable insights into the association between starch degradation, the dynamics of NSC accumulation, remobilization, and grain filling. It thereby provides the potential to optimize NSC dynamics for rice improvement, especially in the large panicle varieties, which generally have asynchronous grain filling problems.

## Data Availability Statement

The original contributions presented in the study are included in the article/Supplementary Material, further inquiries can be directed to the corresponding author/s.

## Author Contributions

MD and DiY conceived the research. XS and NO designed experiments. NO, XS, YT, ZS, DoY, HL, CL, LL, LJ, and BZ performed the experiments and analyzed the data. NO and XS wrote the manuscript. All authors read and approved the final manuscript.

## Conflict of Interest

The authors declare that the research was conducted in the absence of any commercial or financial relationships that could be construed as a potential conflict of interest.

## Supplementary Material

Supplementary Figure 1 The number of grains per panicle **(A)** and seed-setting rate of rice plants **(B)**.

Supplementary Table 1 The period of rice growth at Changsha during the summer season.

Supplementary Table 2 The period of rice growth at Sanya during the winter season.

Supplementary Text 1 The cloned sequence the *RAmy1A* gene and promoter fragment of *SAG12*.
