# Supplementary figures and images for: Senescence-Specific Expression of RAmy1A Accelerates Non-structural Carbohydrate Remobilization and Grain Filling in Rice (Oryza sativa L.)
Source: Front Plant Sci. 2021 Apr 27;12:647574. doi: 10.3389/fpls.2021.647574 (PMC8111089; doi:10.3389/fpls.2021.647574)

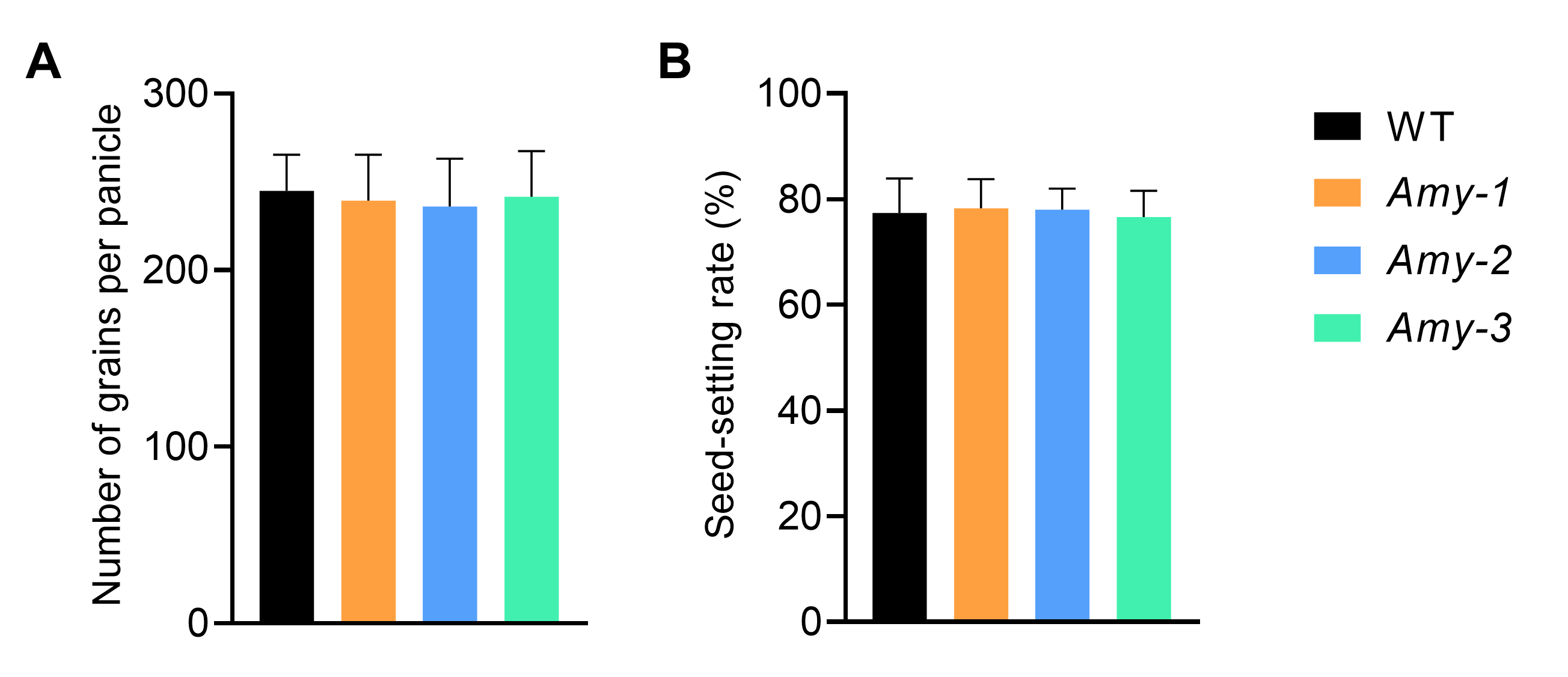

Supplement: Supplementary Figure 1 — The number of grains per panicle (A) and seed-setting rate of rice plants (B). [file Image_1.TIF]
