# Supplementary material for: Senescence-Specific Expression of RAmy1A Accelerates Non-structural Carbohydrate Remobilization and Grain Filling in Rice (Oryza sativa L.)
Source: Front Plant Sci. 2021 Apr 27;12:647574. doi: 10.3389/fpls.2021.647574 (PMC8111089; doi:10.3389/fpls.2021.647574)
Supplement: Supplementary Text 1 — The cloned sequence the RAmy1A gene and promoter fragment of SAG12. [file Data_Sheet_1.DOCX]

The promoter fragment of SAG12 gene:

GATATCTCTTTTTATATTCAAACAATAAGTTGAGATATGTTTGAGAAGAGGACAACTATTCTCGTGGAGCACCGAGTCTGTTTTATATTAGAAACCCGATTGTTATTTTTAGACTGAGACAAAAAAGTAAAATCGTTGATTGTTAAAATTTAAAATTAGTTTCATCACGTTTCGATAAAAAAATGATTAGTTATCATAGCTAATATAGCATGATTCTAAATTTGTTTTTTGACACCCTTTTTTTCTCTCTTTGGTGTTTTCTTAACATTAGAAGAACCCATAACAATGTACGTTCAAATTAATTAAAAACAATATTTCCAAGTTTTATATACGAAACTTGTTTTTTTAATGAAAACAGTTGAATAGTTGATTATGAATTAGTTAGATCAATACTCAATATATGATCAATGATGTATATATATGAACTCAGTTGTTATACAAGAAATGAAAATGCTATTTAAATACCGATCATGAAGTGTTAAAAAGTGTCAGAATATGACATGAAGCGTTTTGTCCTACCGGGTATCGAGTTATAGGTTTGGATCTCTCAAGAATATTTTGGGCCATATTAGTTATATTTGGGCTTAAGCGTTTTGCAAAGAGACGAGGAAGAAAGATTGGGTCAAGTTAACAAAACAGAGACACTCGTATTAGTTGGTACTTTGGTAGCAAGTCGATTTATTTGCCAGTAAAAACTTGGTACACAACTGACAACTCGTATCGTTATTAGTTTGTACTTGGTACCTTTGGTTAAGAAAAAGTTGATATAGTTAAATCAGTTGTGTTCATGAGGTGATTGTGATTTAATTTGTTGACTAGGGCGATTCCTTCACATCACAATAACAAAGTTTTATAGATTTTTTTTTATAACATTTTTGCCACGCTTCGTAAAGTTTGGTATTTACACCGCATTTTTCCCTGTACAAGAATTCATATATTATTTATTTATATACTCCAGTTGACAATTATAAGTTTATAACGTTTTTACAATTATTTAAATACCATGTGAAGATCCAAGAATATGTCTTACTTCTTCTTTGTGTAAGAAAACTAACTATATCACTATAATAAAATAATTCTAATCATTATATTTGTAAATATGCAGTTATTTGTCAATTTTGAATTTAGTATTTTAGACGGTTATCACTTCAGCCAAATATGATTTGGATTTAAGTCCAAAATGCAATTTCGTACGTATCCCTCTTGTCGTCTAATGATTATTTCAATATTTCTTATATTATCCCTAACTACAGAGCTACATTTATATTGTATTCTAATGACAGGGAAACTTTCATAGAGATTCAGATAGATGAAATTGGTGGGAAACATCATTGAACAGGAAACTTTTAGCAAATCATATCGATTTATCTACAAAAGAATACTTAGCGTAATGAAGTTCACTTGTTGTGAATGACTATGATTTGATCAAATTAGTTAATTTTGTCGAATCATTTTTCTTTTTGATTTGATTAAGCTTTTAACTTGCACGAATGGTTCTCTTGTGAATAAACAGAATCTTTGAATTCAAACTATTTGATTAGTGAAAAGACAAAAGAAGATTCCTTGTTTTTATGTGATTAGTGATTTTGATGCATGAAAGGTACCTACGTACTACAAGAAAAATAAACATGTACGTAACTACGTATCAGCATGTAAAAGTATTTTTTTCCAAATAATTTATACTCATGATAGATTTTTTTTTTTTGAAATGTCAATTAAAAATGCTTTCTTAAATATTAATTTTAATTAATTAAATAAGGAAATATATTTATGCAAAACATCATCAACACATATCCAACTTCGAAAATCTCTATAGTACACAAGTAGAGAAAATAAATTTTACTAGATACAAACTTCCTAATCATCAATTATAAATGTTTACAAAACTAATTAAACCCACCACTAAAATTAACTAAAAATCCGAGCAAAGTGAGTGAACAAGACTTGATTTCAGGTTGATGTAGGACTAAAATGGCTACGTATCAAACATCAACGATCATTTAGTTATGTATGAATGAATGTAGTCATTACTTGTAAAACAAAAATGCTTTGATTTGGATCAATCACTTCATGTGAACATTAGCAATTACATCAACCTTATTTTCACTATAAAACCCCATCTCAGTACCCTTCTGAAGTAATCAAATTAAGAGCAAAAGTCATTTAACTTTCCTAAAACC

The sequence of the cloned RAmy1A gene:

ATGCAGGTGCTGAACACCATGGTGAACAAACACTTCTTGTCCCTTTCGGTCCTCATCGTCCTCCTTGGCCTCTCCTCCAACTTGACAGCCGGGCAAGTCCTGTTTCAGGGATTCAACTGGGAGTCGTGGAAGGAGAATGGCGGGTGGTACAACTTCCTGATGGGCAAGGTGGACGACATCGCCGCAGCCGGCATCACCCACGTCTGGCTCCCTCCGCCGTCTCACTCTGTCGGCGAGCAAGGCTACATGCCTGGGCGGCTGTACGATCTGGACGCGTCTAAGTACGGCAACGAGGCGCAGCTCAAGTCGCTGATCGAGGCGTTCCATGGCAAGGGCGTCCAGGTGATCGCCGACATCGTCATCAACCACCGCACGGCGGAGCACAAGGACGGCCGCGGCATCTACTGCCTCTTCGAGGGCGGGACGCCCGACTCCCGCCTCGACTGGGGCCCGCACATGATCTGCCGCGACGACCCCTACGGCGATGGCACCGGCAACCCGGACACCGGCGCCGACTTCGCCGCCGCGCCGGACATCGACCACCTCAACAAGCGCGTCCAGCGGGAGCTCATTGGCTGGCTCGACTGGCTCAAGATGGACATCGGCTTCGACGCGTGGCGCCTCGACTTCGCCAAGGGCTACTCCGCCGACATGGCAAAGATCTACATCGACGCCACCGAGCCGAGCTTCGCCGTGGCCGAGATATGGACGTCCATGGCGAACGGCGGGGACGGCAAGCCGAACTACGACCAGAACGCGCACCGGCAGGAGCTGGTCAACTGGGTCGATCGTGTCGGCGGCGCCAACAGCAACGCCACGGCGTTCGACTTCACCACCAAGGGCATCCTCAACGTCGCCGTGGAGGGCGAGCTGTGGCGCCTCCGCGGCGAGGACGGCAAGGCGCCCGGCATGATCGGGTGGTGGCCGGCCAAGGCGACGACCTTCGTCGACAACCACGACACCGGCTCGACGCAGCACCTGTGGCCGTTCCCCTCCGACAAGGTCATGCAGGGCTACGCATACATCCTCACCCACCCCGGCAACCCATGCATCTTCTACGACCATTTCTTCGATTGGGGTCTCAAGGAGGAGATCGAGCGCCTGGTGTCAATCAGAAACCGGCAGGGGATCCACCCGGCGAGCGAGCTGCGCATCATGGAAGCTGACAGCGATCTCTACCTCGCGGAGATCGATGGCAAGGTGATCACAAAGATTGGACCAAGATACGACGTCGAACACCTCATCCCCGAAGGCTTCCAGGTCGTCGCGCACGGTGATGGCTACGCAATCTGGGAGAAAATCTGA
